# Evaluation of Anti-Inflammatory Potential of the New Ganghwaljetongyeum on Adjuvant-Induced Inflammatory Arthritis in Rats

**DOI:** 10.1155/2016/1230294

**Published:** 2016-06-13

**Authors:** Wangin Kim, Sangbin Park, Chanhun Choi, Youg Ran Kim, Inkyu Park, Changseob Seo, Daehwan Youn, Wook Shin, Yumi Lee, Donghee Choi, Mirae Kim, Hyunju Lee, Seonjong Kim, Changsu Na

**Affiliations:** ^1^College of Korean Medicine, Dongshin University, 185 Geonjae-ro, Naju-si, Jeollanam-do 58245, Republic of Korea; ^2^College of Pharmacy, Chonnam National University, 77 Yongbong-ro, Buk-gu, Gwangju 61186, Republic of Korea; ^3^College of Medicine, Chonnam National University, 77 Yongbong-ro, Buk-gu, Gwangju 61186, Republic of Korea; ^4^Mibyeong Research Center, Korea Institute of Oriental Medicine, 1672 Yuseong-daero, Yuseong-gu, Daejeon 34054, Republic of Korea; ^5^School of Information and Communications, Gwangju Institute of Science and Technology, 123 Cheomdangwagi-ro, Buk-gu, Gwangju 61005, Republic of Korea

## Abstract

Ganghwaljetongyeum (GHJTY) has been used as a standard treatment for arthritis for approximately 15 years at the Korean Medicine Hospital of Dongshin University. GHJTY is composed of 18 medicinal herbs, of which five primary herbs were selected and named new Ganghwaljetongyeum (N-GHJTY). The purpose of the present study was to observe the effect of N-GHJTY on arthritis and to determine its mechanism of action. After confirming arthritis induction using complete Freund's adjuvant (CFA) in rats, N-GHJTY (62.5, 125, and 250 mg/kg/day) was administered once a day for 10 days. In order to determine pathological changes, edema of the paws and weight were measured before and for 10 days after N-GHJTY administration. Cytokine (TNF-*α*, IL-1*β*, and IL-6) levels and histopathological lesions in the knee joint were also examined. Edema in the paw and knee joint of N-GHJTY-treated rats was significantly decreased at 6, 8, and 10 days after administration, compared to that in the CFA-control group, while weight consistently increased. Rats in N-GHJTY-treated groups also recovered from the CFA-induced pathological changes and showed a significant decline in cytokine levels. Taken together, our results showed that N-GHJTY administration was effective in inhibiting CFA-induced arthritis via anti-inflammatory effects while promoting cartilage recovery by controlling cytokine levels.

## 1. Introduction

Arthritis collectively refers to more than 100 rheumatic diseases that are characterized by inflammation, pain, and stiffness in the musculoskeletal system and that range from localized, self-limiting conditions to systemic, autoimmune processes [1]. Arthritis occurs in all age groups and peaks between the ages of 35 and 50, affecting ~1% of the world's population [1, 2]. Rheumatoid arthritis (RA) is a systemic inflammatory disease that attacks the joints by producing proliferative synovitis, leading to destruction of articular cartilage and underlying bone. RA affects approximately 0.5% to 1% adults worldwide, with women being affected two to three times more frequently than men; RA is also associated with a high mortality rate [1, 3].

Autoimmunity and chronic inflammation are activated by an imbalance between pro- and anti-inflammatory cytokines, thereby causing joint damage in RA [4]. RA is characterized by angiogenesis in the synovial membrane, which contributes to the advancement of the disease, as well as the production of inflammatory cells that infiltrate and destroy the synovial tissue [1, 5, 6]. In RA, production of both cytokines and chemokines is induced by macrophage- and fibroblast-derived cytokines [7]. Of these, proinflammatory interleukin 1 beta (IL-1*β*) and tumor necrosis factor alpha (TNF-*α*) are often targeted in RA treatment strategies [8, 9].

Our previous study showed that Ganghwaljetongyeum (GHJTY) can effectively attenuate RA by inhibiting synovial cell proliferation as well as the production of proinflammatory mediators from macrophage-like cells [10]. However, 18 medicinal herbs are included in GHJTY, making it impractical for clinical application. In order to improve the efficacy and convenience of pharmaceutically prescribing GHJTY, we used bioinformatics (http://combio.gist.ac.kr/herding) [11] to select the five medicinal herbs with the greatest potential to treat arthritis. The selected medicinal herbs were* Ostericum koreanum* Maximowicz (Osterici Radix, OK),* Lonicera japonica* Thunberg (Lonicerae Folium, LJ),* Clematis mandshurica* Ruprecht (Clematis Radix, CM),* Angelica gigas* Nakai (Angelicae Gigantis Radix, AG), and* Phellodendron amurense* Ruprecht (Phellodendri Cortex, PA).

A review of the literature revealed that each of the selected herbs is effective in targeting certain aspects of RA pathophysiology. For example, OK inhibits the production of inflammatory mediators by downregulating nuclear factor kappa beta (NF-*κ*B) and mitogen-activated protein kinase (MAPK) activity in lipopolysaccharide-stimulated RAW264.7 cells [12]. Additionally, anti-inflammatory activity of the major constituents of LJ has been shown [13], along with the ability of CM extract to interact with NF-*κ*B, TNF-*α*, and cyclooxygenase 2 (COX-2) in rats with collagen-induced arthritis [14]. Studies have also shown that AG inhibits focal and systemic inflammation in dinitrofluorobenzene-induced inflammation models and that PA protects against human osteoarthritis by regulating aggrecanases, matrix metalloproteinases (MMPs)/tissue inhibitors of metalloproteinases (TIMP), proinflammatory cytokines, and MAPK pathway signaling [15, 16]. However, the effects of combining these herbal medicines remain unclear.

The purpose of the present study was to evaluate the efficacy and mechanism of action of this new GHJTY (N-GHJTY) using an animal model of RA. Specifically, we used rats with complete Freund's adjuvant- (CFA-) induced arthritis as an experimental animal model used to mimic human RA, as it produces profound systemic inflammation that results in severe joint swelling and remodeling [17]. Moreover, CFA is typically used to investigate therapeutic agents with antiarthritic potential [18]. Our specific aims were to (1) confirm the quality assessment of marker components in N-GHJTY, (2) determine the antiarthritic effects of N-GHJTY by measuring paw edema and observing gross lesions of the paw and knee joint, (3) evaluate potential adverse effects of N-GHJTY by histopathological investigation, and (4) assess the effect of N-GHJTY on proinflammatory cytokines by examining levels of TNF-*α*, IL-1*β*, and IL-6.

## 2. Materials and Methods

### 2.1. Animals

Adult male Sprague-Dawley rats, weighing 200–210 g, were housed in a room with constant temperature (24–26°C) and humidity (40–60%). Food (Pellet, GMO, Korea) and water were available ad libitum. Animals were acclimated to the laboratory environment for 1 week before the experiment, and all procedures were approved by the Institutional Animal Care and Use Committee of the Dongshin University (2014-03-02).

### 2.2. CFA-Induced Arthritis and Drug Administration

In the primary adjuvant-induced arthritis model, 0.25 mL of CFA (Sigma, St. Louis, MD, USA) was injected into the left hind knee joint. After 7 days, secondary arthritis was induced by injecting 0.05 mL of CFA under the left hind knee joint and left hind sole. Animals were then divided into the following five groups (*n* = 6/group): normal, CFA arthritis (CFA-control) and CFA arthritis treated with 62.5, 125, and 250 mg/kg of N-GHJTY extract per day (N-GHJTY-62.5, N-GHJTY-125, and N-GHJTY-250, resp.). Oral administration of N-GHJTY was initiated on the 10th day after arthritis induction and continued for 10 days thereafter. Animals were anesthetized using 2.5% isoflurane and volumes of the hind paw and knee joint were measured using a Digital Plethysmometer (LE7500, Panlab, Spain) 0, 2, 4, 6, 8, and 10 days after oral N-GHJTY administration.

### 2.3. Preparation of Herbal Materials

The five herbal medicines forming N-GHJTY were purchased from Omniherb Co. (Yeongcheon, Korea), and their origin was taxonomically confirmed by Professor Jong-Kil Jeong in the Department of Herbology at the College of Oriental Medicine, Dongshin University.

The five herbs (OK, LJ, AG, CM, and PA) were combined in a 6 : 4 : 4 : 4 : 3 ratio. N-GHJTY was prepared via water extraction at 100°C for 3 h and concentrated using a rotary vacuum evaporator (EYELA, Japan) after filtration followed by lyophilization using a vacuum freeze drier (Samwon Freezing Engineering Co., Korea). The yield was about 29.5%.

### 2.4. Reagents and High-Performance Liquid Chromatography (HPLC) Analysis

**Figure 1:**
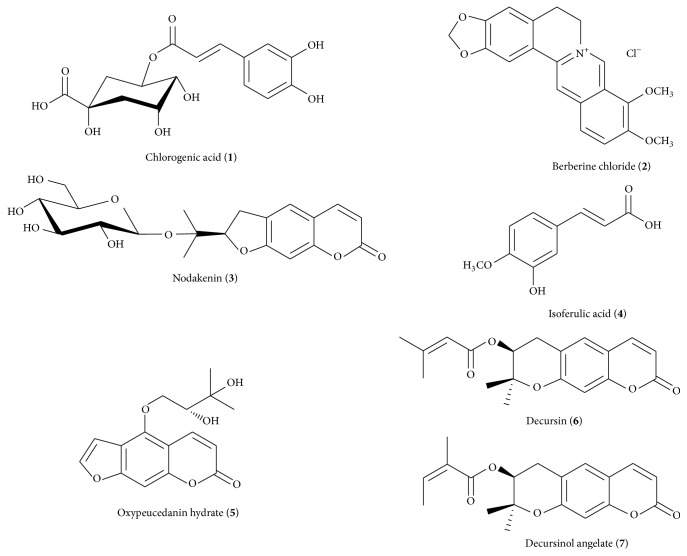
Chemical structure of the seven marker compounds.

Chlorogenic acid (**1**) and berberine chloride (**2**) were purchased from Acros Organics (Pittsburgh, PA, USA) and Sigma-Aldrich Co. LLC. (St. Louis, MO, USA), respectively. Nodakenin (**3**), decursin (**6**), and decursinol angelate (**7**) were purchased form NPC BioTechnology (Yeongi, Korea). Isoferulic acid (**4**) and oxypeucedanin hydrate (**5**) were purchased from ChemFaces Biochemical Co. Ltd. (Wuhan, China). The purities of the seven reference compounds were ≥98.0% by HPLC analysis and the chemical structures of the seven marker compounds are shown in Figure 1. HPLC-grade solvents, methanol, acetonitrile, and water were obtained from J.T.Baker (Phillipsburg, NJ, USA). Analytical grade formic acid was purchased from Sigma-Aldrich (St. Louis, MO, USA).

For quality assessment of the seven marker compounds in N-GHJTY, all experiments were conducted using a Shimadzu Prominence LC-20A Series (Shimadzu, Kyoto, Japan) equipped with a solvent delivery unit (LC-20AT), online degasser (DGU-20A3), column oven (CTO-20A), autosample injector (SIL-20AC), and photodiode array (PDA) detector (SPD-M20A). Lab solution software (version 5.54 SP3, Kyoto, Japan) was used for data acquisition and processing. Chromatographic separation of all analytes was performed using a Waters SunFire C18 column (4.6 × 250 mm; 5 *μ*m, Milford, MA, USA). The mobile phases consisted of 0.1% (v/v) formic acid in distilled water (A) and 0.1% (v/v) formic acid in acetonitrile (B) and the gradation condition was optimized as follows: with range of 0–30 min, 10–100% B; 30–40 min, 100% B; 40–50 min, 100–10% B; 50–60 min, 10% B. The flow rate of mobile phase was maintained at 1.0 mL/min, and the injection volume was 10 mL. The flow rate was maintained at 1.0 mL/min, and the injection volume was 10 mL. For HPLC analysis, lyophilized N-GHJTY (200 mg) was dissolved in 20 mL of 70% methanol and extracted for 60 min by sonication. The N-GHJTY extract solution was passed through a 0.2-*μ*m syringe filter (PALL Life Sciences, Ann Arbor, MI, USA) before HPLC analysis.

### 2.5. Blood and Serum Tests

Blood samples were collected, and 100 *μ*L was used for a complete blood count (CBC) analysis via a Multispecies Hematology Analyzer (950, Hemavet, USA). Serum was separated from the rest of the blood using a high-speed centrifuge (VS-600CFi, Korea) at 3500 rpm (*g* = 27.391) for 20 min, and aspartate aminotransferase (AST) and alanine transaminase (ALT) levels were measured.

### 2.6. Measurement of TNF-*α*, IL-1*β*, and IL-6

TNF-*α* was measured using a Rat TNF-*α* kit (Invitrogen, USA), IL-1*β* was assessed using a Rat IL-1*β* kit (R&D Systems, USA), and IL-6 was evaluated using a Rat IL-6 kit (Invitrogen, USA). Optical densities (OD) of all samples were measured at 450 nm via Spectramax (M2, Molecular Devices, USA).

### 2.7. Hematoxylin and Eosin (HE) Staining

The right knee joint was removed and fixed in Bouin solution for over 24 h. Decalcification was conducted in a 2.5% nitric acid solution, which was changed once a day for 7 days. The removed tissue was dehydrated using Tissue Processor (Tissue-Tex II, Japan), deparaffinized, stained with HE (Muto, Japan), and observed under an optical microscope (Nikon, Japan).

### 2.8. Safranin O-Fast Stain

After deparaffinization, the right knee joint was reacted with Weigert's Iron Hematoxylin (Sigma, USA) solution for 10 min and stained with 0.001% Fast Green (Sigma, USA) solution for 5 min. The knee joint tissue was then reacted with 1% acetate solution for 10 s and stained with 0.1% Safranin O (Sigma, USA) solution for 5 min; thereafter, the tissue was dehydrated and observed under an optical microscope (Nikon, Japan).

### 2.9. Statistical Analysis

Data were analyzed using SPSS 21.0 version for Windows by a nonparametric Mann-Whitney *U* test. A one-way analysis of variance was conducted on each group, and results are expressed as mean ± standard error (SE). Comparisons between groups were performed using the post hoc least squared differences (LSD) test. *P* < 0.05 and *P* < 0.01 were considered statistically significant.

## 3. Results

### 3.1. Quality Assessment of Seven Marker Components in N-GHJTY

**Figure 2:**
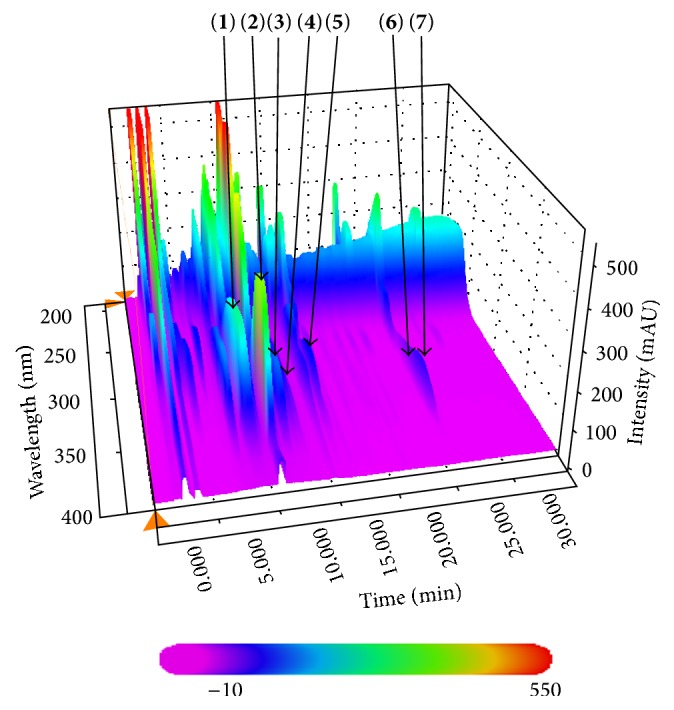
Three-dimensional chromatogram of N-GHJTY. Chlorogenic acid (**1**), berberine chloride (**2**), nodakenin (**3**), isoferulic acid (**4**), oxypeucedanin hydrate (**5**), decursin (**6**), and decursinol angelate (**7**).

HPLC was performed using the seven marker compounds in N-GHJTY for quality control. The selected compounds were as follows: compound** 1** (Lonicerae Folium), compound** 2** (Phellodendri Cortex), compounds** 3**,** 6**, and** 7** (Angelicae Gigantis Radix), compound** 4** (Clematis Radix), and compound** 5** (Osterici Radix). All analytes were separated within 30 min and the typical three-dimensional chromatogram of the 70% methanol extract of N-GHJTY is shown in Figure 2. Quantitation was achieved by photodiode array (PDA) detection at 310 nm (**5**), 325 nm (**1** and** 4**), 330 nm (**6** and** 7**), 335 nm (**3**), and 340 nm (**2**) based on retention time and UV spectrum. The retention times of components** 1**–**7** were 8.94, 10.80, 12.00, 12.86, 15.95, 26.02, and 26.24 min, respectively. Using a calibration curve, we determined that correlation coefficients (*r* ^2^) of all seven compounds were ≥0.9996. Under optimized chromatography conditions, concentrations of N-GHJTY marker compounds** 1**–**7** were 5.46 ± 0.33, 1.87 ± 0.29, 1.70 ± 0.18, 1.64 ± 0.58, 2.03 ± 0.33, 1.09 ± 0.02, and 0.81 ± 0.01 mg/g, respectively.

### 3.2. Effect of N-GHJTY on Gross Lesions of the Paw and Knee Joint

**Figure 3:**
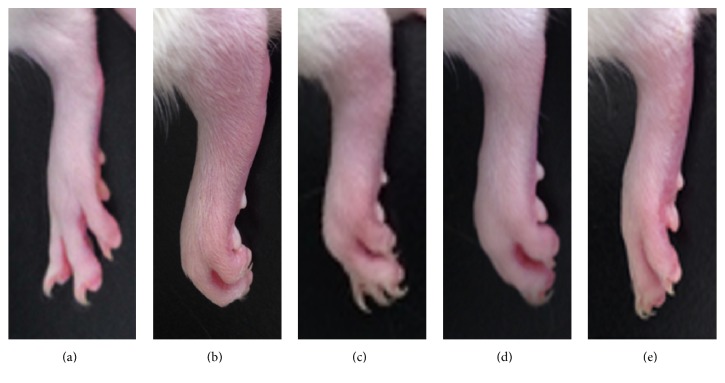
Effect on N-GHJTY in gross lesions in the hind paw and knee swelling in rats with CFA-induced arthritis. (a) Normal group, (b) CFA-control group, (c) N-GHJTY-62.5 group (62.5 mg/kg), (d) N-GHJTY-125 group (125 mg/kg), and (e) N-GHJTY-250 group (250 mg/kg).

Paw and knee joint swelling and rubefaction served as external objective indicators for evaluating the severity of the inflammatory arthritic model. The CFA-control group showed rubefaction and hind paw and knee joint swelling—both of which gradually decreased following N-GHJTY treatment at all concentrations (62.5, 125, and 250 mg/kg) (Figure 3).

### 3.3. Effect of N-GHJTY on Paw and Knee Joint Swelling

Changes in paw and knee joint swelling are presented in Table 1. Approximately 3 days after the second immunization, the rat knee joint began to swell and the paw and knee joint were observed to increase in size. On the 10th day, the CFA-control group showed a significant increase in both paw and knee joint swellings compared to the normal group. This volume significantly decreased in the N-GHJTY-250 group on the 6th day, the N-GHJTY-125 group on the 8th day, and the N-GHJTY-62.5 group on the 10th day compared to the CFA-control group (Table 1).

### 3.4. Effect of N-GHJTY on Body Weight

Twelve days after CFA injection, statistically significant reductions in body weight were observed in the CFA-control group when compared to the normal group. N-GHJTY treatment at all concentrations (62.5, 125, and 250 mg/kg) resulted in an increase in body weight; however, this change was not significant (Table 2).

### 3.5. Effect of N-GHJTY on Transaminase Levels

Aspartate aminotransferase (AST) and alanine aminotransferase (ALT) levels are indicated in Table 3. As shown in the results, only the N-GHJTY-250 group showed a significant decrease in AST and ALT when compared to CFA-control group rats.

### 3.6. Effect of N-GHJTY on Proinflammatory Cytokines

TNF-*α*, IL-1*β*, and IL-6 levels are indicated in Table 4. As shown in the results, TNF-*α*, IL-1*β*, and IL-6 in the CFA-control group showed a significant increase when compared to levels in the normal group rats. A significant decrease in TNF-*α*, IL-1*β*, and IL-6 levels was observed in all N-GHJTY treatment groups (62.5, 125, and 250 mg/kg) when compared to the CFA-control group rats (Table 4).

### 3.7. Effects of N-GHJTY on Histopathological Changes Assessed with HE Staining

**Figure 4:**
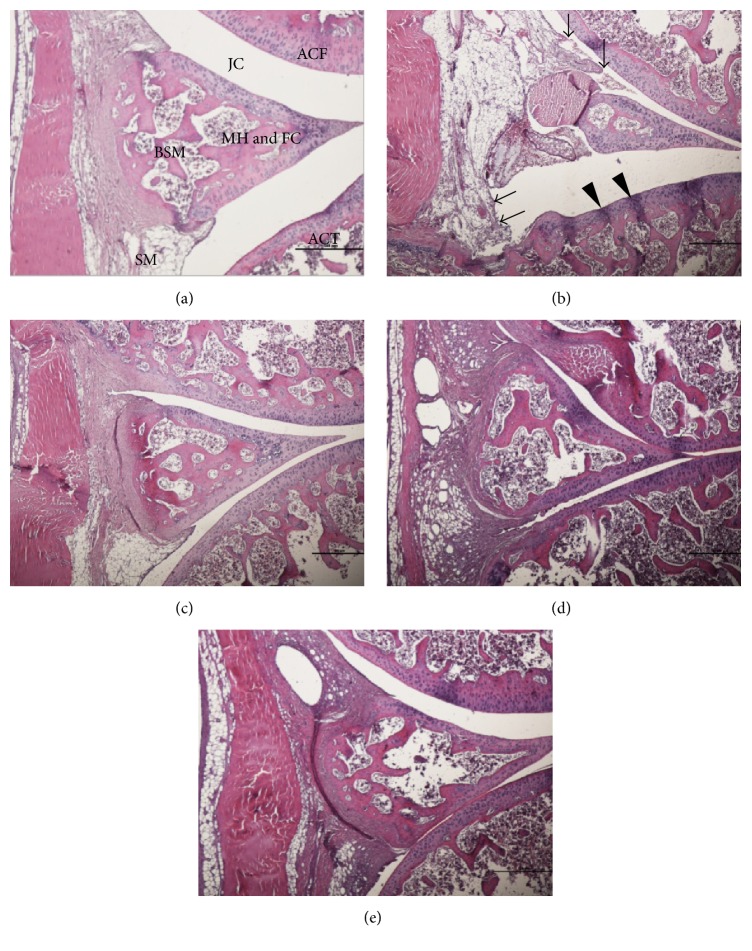
Influence of N-GHJTY on CFA-induced histopathological changes in the knee joints of CFA-induced arthritic rats. Arrows (↓) indicate a damaged synovial membrane. Arrow heads (▼) indicate compressed articular cartilage in the CFA control. (a) Normal group, (b) CFA-control group, (c) N-GHJTY-62.5 group (62.5 mg/kg), (d) N-GHJTY-125 group (125 mg/kg), and (e) N-GHJTY-250 group (250 mg/kg). JC: joint cavity. ACF: articular cartilage of the femur. ACT: articular cartilage of the tibia. BSM: bony spicule within the meniscus. SM: synovial membrane. MH&FC: meniscus of hyaline and fibrocartilage. HE stain, scale bars = 500 nd.

Representative HE stained histopathological lesions in the hind knee joint of normal, CFA-control, N-GHJTY-62.5, N-GHJTY-125, and N-GHJTY-250 groups are shown in Figure 4. Loose synovial membrane, membrane destruction, disorganized cell arrangement, and compressed cartilage were observed in the CFA-control group (Figure 4(b)). Histopathological changes improved in all N-GHJTY groups (62.5, 125, and 250 mg/kg) compared to the CFA-control group. These groups presented close synovial membrane and regular cartilage; the surface of the cartilage in the tibia and femur was smooth and exhibited no noticeable damage (Figures 4(c), 4(d), and 4(e)).

### 3.8. Effects of N-GHJTY on Histopathological Changes Assessed with Safranin O-Fast Staining

**Figure 5:**
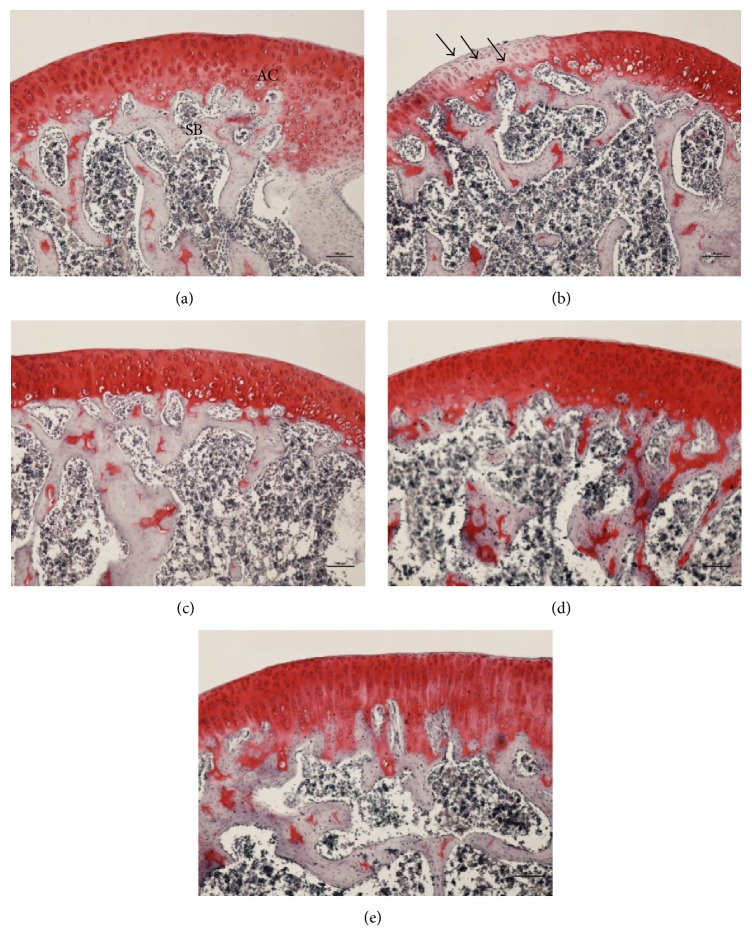
Influence of N-GHJTY on CFA-induced histopathological changes in the knee joints of CFA-induced arthritic rats. A number of shrunken nuclei (arrows ↓) were observed in the CFA control. (a) Normal group, (b) CFA-control group, (c) N-GHJTY-62.5 group (62.5 mg/kg), (d) N-GHJTY-125 group (125 mg/kg), and (e) N-GHJTY-250 group (250 mg/kg). AC: articular cartilage. SB: spongy bone. Safranin O-fast stain, scale bars = 100 *μ*m.

Safranin O-fast stain was conducted to observe histopathological changes in the knee joint. In the normal group (Figure 5(a)), a positive reaction to proteoglycans in the calcified zone was observed and the cartilage was even. The CFA-control group exhibited little positive reactions, and chondrocyte nuclei appeared contracted when compared to nuclei in the normal group (Figure 5(b)). N-GHJTY-62.5, N-GHJTY-125, and N-GHJTY-250 groups exhibited a greater number of positive reactions in the calcified zone of the cartilage when compared to the CFA-control group (Figures 5(c), 5(d), and 5(e)).

## 4. Discussion

RA is an abnormal autoimmune disease that causes synovial inflammation and damage to joint structure. One characteristic that is specific to RA is the network of new blood vessels that extensively develops in the synovial membrane. This destructive vascular tissue, which is called pannus, extends from the synovium to invade the junction between the cartilage and subchondral bone. With progression of the disease, joint inflammation and the resulting structural changes caused by pannus lead to reduced joint motion, possible ankyloses, joint instability, muscle atrophy from disuse, stretching of the ligaments, and involvement of the tendons and muscles [1].

The CFA approach developed by Pearson [19] is a widely used arthritic model, which is induced in susceptible strains of rats via injection of heat-killed mycobacterium tuberculosis [20]. After CFA injection, a rapid, reliable, robust, and easily measurable polyarthritis develops [21]. Importantly, the joint pathology seen in the rat model shares the synovial hyperplasia and cartilage degradation observed in human arthritis, particularly RA [22–24]. CFA has recently become a popular tool to observe the efficacy of herbal medicines for treating arthritis. For example, Zhang et al. used the CFA approach to demonstrate the antiarthritic effect of an extract from* Dioscorea zingiberensis* C.H. Wright [25]. Additionally, Zhang et al. used CFA to demonstrate antioxidant effects of Genkwa flos flavonoids [26], while Obiri et al. demonstrated that* Xylopia aethiopica* (Annonaceae) fruit extract suppresses adjuvant-induced arthritis in rats [27]. Several studies have also shown that herbal medicines are effective in treating RA in humans. Shao Li et al. reported that Qing-Luo-Yin extract may have a protective effect against excessive tissue breakdown, angiogenesis, and degradation of extracellular matrix in RA [28]. Chi Zhang et al. also reported that some herbal medicines have beneficial effects on pain management and swollen joint relief in individuals with RA [29]. Additionally, Liu et al. reported that Xinfeng, a patent Chinese herbal medicine, is effective and safe in the treating RA [30].

In the current study, we found that treatment with the N-GHJTY resulted in a gradual decrease of CFA-induced paw and knee joint swelling, as well as rubefaction. This finding was present in all N-GHJTY-treated groups (62.5, 125, and 250 mg/kg) and exhibited a dose-dependent effect—with the N-GHJTY-250 group showing the greatest decrease in paw swelling (Figure 3). In terms of time course, the N-GHJTY-250 group showed significantly less paw and knee joint swelling on the 6th day after treatment, the N-GHJTY-125 group on the 8th day, and the N-GHJTY-62.5 group on the 10th day (Table 1). Moreover, rats in all N-GHJTY groups (62.5, 125, and 250 mg/kg) exhibited increases in body weight when compared to those in the CFA-control group.

Regarding proinflammatory cytokines, all N-GHJTY-treated groups (62.5, 125, and 250 mg/kg) displayed significant reductions in TNF-*α*, IL-1*β*, and IL-6 when compared to rats in the CFA-control group. RA is initiated by a T cell-mediated immune response that stimulates the release of cytokines and promotes antibody formation, which leads to destruction of the joint [1]. Specifically, TNF-*α* induces the production of IL-1*β* and IL-6 [31], with the former being a crucial mediator of the inflammatory response, causing various autoinflammatory syndromes [32, 33], and the latter being secreted by T cells and macrophages to stimulate the immune response [34, 35]. Thus, proinflammatory cytokines (particularly IL-1*β*, IL-6, and TNF-*α*) play an important role in arthritis onset [36], while inhibitors of these cytokines are effective in controlling chronic inflammation [37, 38].

The protective effects of N-GHJTY were confirmed by our histopathological investigations with HE and Safranin O-fast staining. Specifically, rats in the CFA-control group exhibited loose synovial membrane, membrane destruction, disorganized cell arrangement, and compressed cartilage—all of which were prevented in rats treated with N-GHJTY. As mentioned, release of enzymes and inflammatory mediators from damaged tissues perpetuates the inflammatory process [1]. It has also been shown that cytokines released by inflamed synovial tissue can reach systemic circulation and act on other organs [39]. Rheumatoid factor (RF), which is an autoantibody found in RA, forms an immune complex with immunoglobulin G that contributes to RA progression by further triggering inflammatory responses and attracting inflammatory cells. Thus, the ability of N-GHJTY to protect against cartilage and synovial membrane destruction could additionally prevent systemic inflammation by preventing damaged synovial tissue from releasing cytokines.

To investigate if repeated N-GHJTY treatment could induce toxicity, we observed transaminase levels (AST and ALT) in all N-GHJTY-treated groups. Our findings revealed that N-GHJTY treatment did not alter either AST or ALT, suggesting that N-GHJTY was not toxic to rats. Moreover, investigations of various diseases (including arthritis) have reported that AST and ALT leak into the blood stream in proportion to the extent of tissue damage [40–42]. Indeed, transaminase levels were slightly increased in the CFA-control group in the current study. Consistent with our findings on the anti-inflammatory effects of N-GHJTY, we observed that the highest dose of this new herbal combination (i.e., 250 mg/kg) resulted in a slight decrease of transaminase levels, suggesting improved liver function in these animals.

Taken together, our findings suggest that N-GHJTY administration could prevent inflammatory cells from infiltrating and destroying synovial tissue and could also suppress the release of proinflammatory cytokines. Moreover, N-GHJTY was effective in preventing the destruction of joint tissue and facilitated the repair of CFA-induced injury to the joint cartilage, resulting in reduced paw and knee swelling. Thus, the present study provides evidence supporting the clinical use of N-GHJTY for treating arthritis.

## 5. Conclusions

N-GHJTY, a new complex herbal medication, was effective in treating a rat model of inflammatory arthritis. Specifically, N-GHJTY significantly suppressed the progression of CFA-induced arthritis, as was evident from the decrease in paw and knee joint swelling, and was effective in preventing articular cartilage and synovial tissue degeneration. We also revealed that the protective mechanisms of N-GHJTY treatment could be partially explained by a decrease in the proinflammatory cytokines, TNF-*α*, IL-1*β*, and IL-6. Additional studies are required to determine other molecular mechanisms associated with N-GHJTY administration, as well as specific therapeutic effects.

## Figures and Tables

**Table 1:** Changes in paw swelling after N-GHJTY administration in rats with CFA-induced arthritis (mL).

Group	Before	Days after CFA (days N-GHJTY administration)					
		10 (0)	12 (2)	14 (4)	16 (6)	18 (8)	20 (10)
Normal	1.36 ± 0.03	1.47 ± 0.04	1.49 ± 0.04	1.55 ± 0.05	1.57 ± 0.06	1.63 ± 0.05	1.66 ± 0.06
CFA-control	1.24 ± 0.01	3.77 ± 0.25^##^	3.78 ± 0.26^##^	3.91 ± 0.26^##^	3.91 ± 0.28^##^	3.96 ± 0.28^##^	4.00 ± 0.30^##^
N-GHJTY-62.5	1.26 ± 0.03	3.68 ± 0.33	3.72 ± 0.31	3.68 ± 0.26	3.53 ± 0.23	3.35 ± 0.21	3.23 ± 0.17^*∗*^
N-GHJTY-125	1.24 ± 0.03	3.68 ± 0.15	3.67 ± 0.15	3.60 ± 0.17	3.36 ± 0.20	3.15 ± 0.20^*∗*^	3.03 ± 0.20^*∗*^
N-GHJTY-250	1.31 ± 0.04	3.63 ± 0.16	3.66 ± 0.16	3.49 ± 0.10	3.22 ± 0.08^*∗*^	3.05 ± 0.05^*∗*^	2.91 ± 0.09^*∗∗*^

Mean ± SE.

^##^ *P* < 0.01 versus normal group.

^*∗∗*^ *P* < 0.01 and ^*∗*^ *P* < 0.05 versus CFA-control group.

**Table 2:** Changes in body weight after N-GHJTY administration in rats with CFA-induced arthritis (%).

Group	Before	Days after CFA (days N-GHJTY administration)					
		10 (0)	12 (2)	14 (4)	16 (6)	18 (8)	20 (10)
Normal	100.0 ± 0.0	145.2 ± 1.8	148.3 ± 2.4	153.6 ± 2.8	159.4 ± 3.2	163.4 ± 3.6	164.0 ± 3.7
CFA-control	100.0 ± 0.0	134.8 ± 1.7	139.5 ± 2.2^#^	144.7 ± 3.4	148.9 ± 4.3	153.4 ± 5.2	154.0 ± 5.1
N-GHJTY-62.5	100.0 ± 0.0	135.5 ± 2.2	142.9 ± 1.8	149.6 ± 1.9	157.4 ± 2.2	161.4 ± 1.6	162.1 ± 1.7
N-GHJTY-125	100.0 ± 0.0	136.0 ± 2.5	142.0 ± 2.3	148.6 ± 2.5	156.1 ± 3.2	159.9 ± 3.6	161.1 ± 3.6
N-GHJTY-250	100.0 ± 0.0	136.4 ± 3.5	143.2 ± 3.7	149.5 ± 4.1	157.6 ± 4.8	161.5 ± 5.1	162.8 ± 5.4

Mean ± SE.

^#^ *P* < 0.05 versus normal group.

**Table 3:** Effect of N-GHJTY on aspartate and alanine aminotransferase levels in rats with CFA-induced arthritis.

Group	Aspartate aminotransferase (U/L)	Alanine aminotransferase (U/L)
Normal	82.0 ± 5.0	28.4 ± 1.6
CFA-control	93.7 ± 4.6^#^	38.2 ± 2.9^#^
N-GHJTY-62.5	86.0 ± 7.8	39.0 ± 2.1
N-GHJTY-125	85.4 ± 1.9	28.7 ± 3.4
N-GHJTY-250	80.0 ± 3.0^*∗*^	28.8 ± 1.3^*∗*^

Mean ± SE.

^#^ *P* < 0.05 versus normal group.

^*∗*^ *P* < 0.05 versus CFA-control group.

**Table 4:** Effect of N-GHJTY on TNF-*α*, IL-1*β*, and IL-6 levels in rats with CFA-induced arthritis.

Group	TNF-*α* (pg/mL)	IL-1*β* (pg/mL)	IL-6 (pg/mL)
Normal	2.33 ± 0.12	22.9 ± 0.95	4.3 ± 0.41
CFA-control	4.85 ± 0.67^##^	36.7 ± 0.94^##^	18.0 ± 0.87^##^
N-GHJTY-62.5	3.24 ± 0.32^*∗*^	26.8 ± 0.84^*∗*^	11.0 ± 0.5^*∗*^
N-GHJTY-125	3.01 ± 0.18^*∗*^	26.2 ± 0.52^*∗∗*^	10.4 ± 0.6^*∗*^
N-GHJTY-250	2.84 ± 0.17^*∗*^	25.4 ± 0.84^*∗∗*^	9.7 ± 0.39^*∗*^

Mean ± SE.

^##^ *P* < 0.01 versus normal group.

^*∗∗*^ *P* < 0.01 and ^*∗*^ *P* < 0.05 versus CFA-control group.
